# Developing a Taijiquan exercise pattern for Chinese older adults: a qualitative study based on the exercise commitment model

**DOI:** 10.3389/fpsyg.2026.1883642

**Published:** 2026-09-07

**Authors:** Sun Xianghao, Kritchapol Arsapakdee, Chairat Choosakul

**Affiliations:** 1 Faculty of Physical Education, Henan Polytechnic University, Jiaozuo, China; 2 Department of Health and Sport Science, Faculty of Education, Mahasarakham University, Maha Sarakham, Thailand

**Keywords:** exercise adherence, exercise commitment, intervention framework, older adults, qualitative research, Tai Chi, Taijiquan (T’ai chi Ch’uan)

## Abstract

**Background:**

Taijiquan is widely practiced by older adults and has been associated with benefits for physical health, psychological wellbeing, balance, cognition, and quality of life. However, the long-term maintenance of Taijiquan exercise remains a challenge, and some older adults discontinue participation during the early stages of practice. Exercise commitment provides a useful psychological framework for understanding continued participation in exercise. However, few studies have systematically developed an intervention framework for strengthening Taijiquan exercise commitment among older adults.

**Objective:**

This study aimed to develop a Taijiquan Exercise Pattern for Chinese Elderly (TEP-CE) based on the Taijiquan Exercise Commitment Scale for Chinese Elderly (TECS-CE) and the Exercise Commitment Model.

**Methods:**

A qualitative research design was employed. Sixteen participants from Jiaozuo City, China, were purposively selected and divided into four groups: Taijiquan administrators, Taijiquan instructors, researchers, and elderly Taijiquan practitioners, with four participants in each group. Semi-structured in-depth interviews were conducted. The interview guide was developed based on the eight-factor TECS-CE framework and was reviewed for content validity by five experts using the Index of Item-Objective Congruence. Interview data were transcribed and analyzed using NVivo 12.0 through open, axial, and selective coding. The analysis identified 80 free nodes and generated core recommendations for the development of the TEP-CE.

**Results:**

The qualitative analysis identified five major components of the TEP-CE: (1) Taijiquan exercise intervention content; (2) audio and self-language intervention for exercisers; (3) leader-to-participant intervention; (4) peer intervention and mutual support among participants; and (5) exercise monitoring and recording. The framework was designed to address the eight factors of the TECS-CE, including want-to commitment, have-to commitment, satisfaction, social constraints, involvement alternatives, personal investment, social support, and involvement opportunities.

**Conclusion:**

The TEP-CE provides a structured, theoretically informed framework for strengthening exercise commitment and supporting sustained Taijiquan participation among Chinese older adults. The framework may provide practical guidance for Taijiquan instructors, community organizations, researchers, and policymakers. Future research should develop, implement, and empirically evaluate a complete intervention program based on the proposed framework.

## 1 Introduction

Population aging has created an increasing need for effective strategies to maintain health, functional independence, and quality of life among older adults. Regular physical activity is an important component of healthy aging, and exercise programs that are safe, accessible, culturally meaningful, and sustainable are particularly important for older populations (World Health Organization, 2018; Lightfoot et al., 2018). Although physical activity is widely recognized as beneficial, participation and long-term adherence remain challenging (Lightfoot et al., 2018). Biological, psychological, social, environmental, and cultural factors may influence whether older adults initiate and maintain regular exercise.

Taijiquan is a traditional Chinese mind–body exercise characterized by slow, controlled, continuous movements, postural control, breathing, and focused attention. It has been practiced widely in China and internationally and is increasingly used as a health-promoting physical activity for older adults (Li, 2016). Previous research has reported that Taijiquan may contribute to public health promotion, psychological well-being, mental health, memory, sleep quality, balance, and fall prevention (Li, 2016; Abbott and Lavretsky, 2013; Wang et al., 2014; Tao et al., 2016; Lo and Lee, 2014; Stevens et al., 2014). The relatively low-impact nature of Taijiquan makes it potentially suitable for older adults and individuals with limitations in physical function (Stevens et al., 2014). However, the availability of health benefits alone does not guarantee sustained participation. Recent systematic reviews have indicated that the long-term effectiveness of Tai Chi interventions depends not only on their physiological benefits but also on participants’ intrinsic motivation, perceived enjoyment, social connectedness, instructor support, and sustained engagement in regular practice (Koren et al., 2021; Leung et al., 2022; Fan et al., 2025). These findings suggest that maintaining long-term participation requires interventions that address both psychosocial and behavioral determinants of exercise adherence. Therefore, understanding the mechanisms that promote sustained participation has become an increasingly important research priority in the development of Tai Chi interventions for older adults.

A central challenge in exercise promotion is therefore not simply how to encourage individuals to begin exercising, but how to support continued participation over time. Older adults may discontinue exercise because of changes in motivation, competing activities, lack of social support, dissatisfaction with the exercise environment, insufficient perceived benefits, physical discomfort, or a lack of opportunities for meaningful participation. Consequently, interventions designed to improve long-term exercise participation should address both the psychological and behavioral mechanisms underlying continued participation. Recent systematic reviews have emphasized that effective exercise adherence interventions should be grounded in behavioral theory and incorporate motivational, social, and environmental strategies to support sustained participation among older adults. Rather than relying solely on exercise prescription, contemporary evidence suggests that interventions integrating goal setting, social support, instructor guidance, individualized planning, and behavioral reinforcement are more effective in promoting long-term exercise adherence (Wu et al., 2025; Shaw et al., 2022). These findings underscore the importance of developing theory-informed intervention frameworks that translate psychological constructs into practical strategies for sustaining exercise behavior.

One construct that has been used to explain continued participation in sport and exercise is commitment (Corbin et al., 1987; Martin and Sinden, n.d.; Scanlan et al., 1993). The Sport Commitment Model conceptualizes commitment as an individual’s desire and resolve to continue participating in a sport or physical activity (Scanlan et al., 1993). Exercise commitment has subsequently been applied to the exercise context and has been associated with exercise behavior and continued participation (Wilson et al., 2004). Commitment is particularly relevant to older adults because continued exercise may depend not only on perceived health benefits but also on personal intention, perceived obligation, satisfaction, social influences, personal investment, social support, and the availability of alternative activities (Scanlan et al., 1993; Wilson et al., 2004).

The Exercise Commitment Scale has been used to examine the psychological factors associated with exercise participation and adherence (Chen et al., 2005; Wilson et al., 2004). Previous work has identified multiple dimensions of exercise commitment and its determinants, including the desire to continue exercising, perceived obligation, satisfaction, social constraints, alternative activities, personal investment, social support, and involvement opportunities (Scanlan et al., 1993; Wilson et al., 2004).

In the context of Taijiquan, the Taijiquan Exercise Commitment Scale for Chinese Elderly (TECS-CE) was developed to assess exercise commitment among older Chinese adults (Sun and Choosakul, 2023). The TECS-CE comprises eight factors and 33 items. Two factors represent the commitment dimension itself—want-to commitment and have-to commitment—while six factors represent determinants of commitment: satisfaction, social constraints, involvement alternatives, personal investment, social support, and involvement opportunities (Sun and Choosakul, 2023). Thus, the TECS-CE provides a theoretically grounded framework for examining both the psychological commitment of older adults and the conditions that may influence their continued participation in Taijiquan.

Although the Exercise Commitment Model has provided a useful theoretical basis for explaining exercise behavior (Scanlan et al., 1993; Sun and Choosakul, 2023; Waldron and Troupe, 2008), previous research has focused primarily on measuring exercise commitment and examining the relationships among commitment-related variables. Relatively little attention has been given to translating these theoretical dimensions into practical intervention strategies that support sustained exercise participation among older adults. In particular, limited evidence is available regarding how commitment-related constructs can be operationalized into structured intervention frameworks for culturally meaningful physical activities such as Taijiquan. Therefore, there remains a need to bridge the gap between exercise commitment theory and practical intervention design for older adult populations.

This issue is particularly important in the Chinese context. Taijiquan is not only an exercise activity but also a culturally embedded traditional practice. In Jiaozuo City, where Taijiquan has substantial cultural and social significance, participation may be influenced by local traditions, community identity, intergenerational transmission, group practice, and the symbolic value of Taijiquan as part of Chinese cultural heritage. Therefore, an intervention framework developed for Chinese older adults should not simply transfer a general Western exercise adherence model into the Chinese context. It should also consider the cultural meaning of Taijiquan, the role of community leaders and instructors, interpersonal relationships among practitioners, and the experiences of older adults themselves.

The present study therefore used a qualitative approach to explore how the eight dimensions of the TECS-CE could be translated into practical intervention strategies (Sun and Choosakul, 2023). Perspectives were obtained from four groups with different relationships to Taijiquan: administrators, instructors, researchers, and older adult practitioners. This multi-perspective design was intended to provide a broader understanding of how an intervention framework could be developed and implemented.

Accordingly, this study aimed to develop a Taijiquan Exercise Pattern for Chinese Elderly (TEP-CE) based on the Exercise Commitment Model and the TECS-CE (Scanlan et al., 1993; Wilson et al., 2004; Sun and Choosakul, 2023). The specific objectives were to: (1) explore recommendations for intervention based on the eight dimensions of the TECS-CE; (2) identify core concepts and recommendations from interviews with multiple stakeholder groups; and (3) synthesize these findings into a structured framework for strengthening Taijiquan exercise commitment and supporting continued participation among Chinese older adults.

## 2 Methods

### 2.1 Research design

This study employed a qualitative research design using purposive sampling and semi-structured, in-depth interviews. A qualitative approach was selected because the study sought to explore the experiences, perceptions, recommendations, and practical perspectives of individuals with extensive knowledge or experience related to Taijiquan exercise among older adults (Lune and Berg, 2017; Creswell and Poth, 2016).

Purposive sampling was used to recruit participants who possessed extensive knowledge and experience relevant to the research objectives. This sampling strategy is appropriate for qualitative research because it enables researchers to intentionally select information-rich participants who can provide comprehensive and meaningful insights into the phenomenon under investigation (Lune and Berg, 2017; Creswell and Poth, 2016).

The study was guided by the Exercise Commitment Model and the eight-factor structure of the Taijiquan Exercise Commitment Scale for Chinese Elderly (TECS-CE). The qualitative data were used to explore how these theoretical dimensions could be translated into practical intervention strategies and subsequently organized into a structured Taijiquan Exercise Pattern for Chinese Elderly (TEP-CE).

### 2.2 Participants and sampling

Participants were recruited from Jiaozuo City, China, using purposive sampling. This sampling strategy was selected to intentionally recruit information-rich participants with knowledge and experience relevant to the research objectives (Lune and Berg, 2017; Creswell and Poth, 2016).

The sample size was determined according to qualitative research principles, in which adequacy is primarily determined by the richness, relevance, and sufficiency of the information obtained rather than by statistical representativeness. Sample adequacy was assessed with consideration of information saturation and information power, including the specificity of the research aim, the characteristics of the participants, and the diversity of stakeholder perspectives (Sun, 2012; Creswell, 1998; Saunders et al., 2018; Malterud et al., 2016).

Participants were recruited from four stakeholder groups with different roles and experiences related to Taijiquan:

- Taijiquan administrators or managers (*n* = 4), who had experience in the management, promotion, or development of Taijiquan-related activities;
- Taijiquan instructors or leaders (*n* = 4), who had extensive experience teaching or leading Taijiquan practice;
- Researchers or scholars (*n* = 4), who had advanced academic qualifications and research experience related to Taijiquan or exercise; and
- Older adult Taijiquan practitioners (*n* = 4), who had personal experience participating in Taijiquan exercise.

The final sample comprised 16 participants.

Eligibility criteria were established according to the participants’ roles and experience. Administrators were required to have more than 5 years of experience in Taijiquan management. Instructors were required to have more than 5 years of experience teaching or leading Taijiquan practice. Researchers were required to hold at least an associate senior academic title and have published authoritative research in the field. Older adult practitioners were required to be 60–74 years of age and have practical experience participating in Taijiquan exercise.

The four-group sampling design was intended to incorporate perspectives from policy and management, practical instruction, academic research, and direct exercise experience, thereby providing comprehensive information for the development of the Taijiquan Exercise Pattern for Chinese Elderly.

### 2.3 Interview instrument

**Figure 1:**
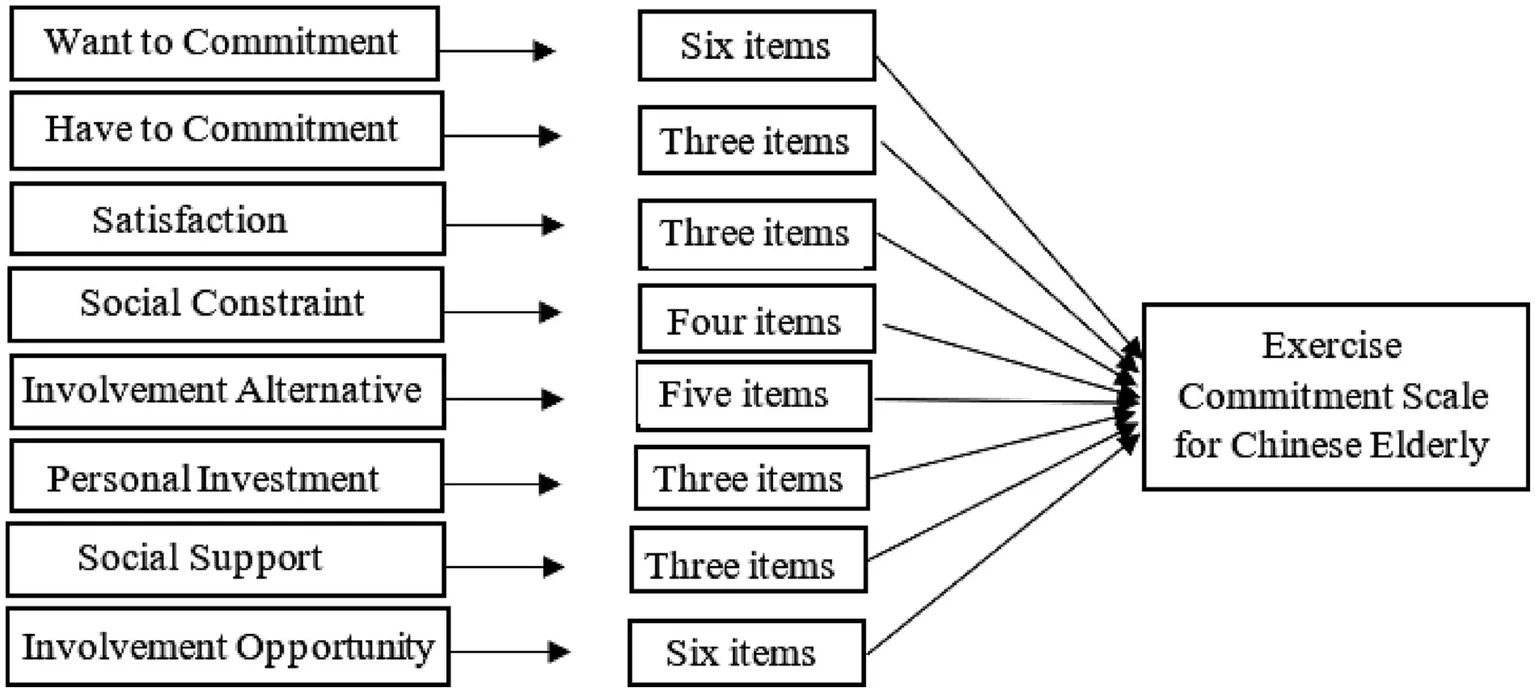
Conceptual framework of the Taijiquan exercise commitment scale for Chinese elderly (TECS-CE).

The interview guide was developed based on the Taijiquan Exercise Commitment Scale for Chinese Elderly (TECS-CE). The TECS-CE comprises 33 items organized into eight factors under two major dimensions: Taijiquan Exercise Commitment, consisting of want-to commitment and have-to commitment, and Determinants of Taijiquan Exercise Commitment, comprising satisfaction, social constraints, involvement alternatives, personal investment, social support, and involvement opportunities. The conceptual framework of the TECS-CE is presented in Figure 1, while the complete list of commitment constructs and subscale items is provided in Appendix A.

Based on this theoretical framework, a semi-structured interview guide was developed to explore how each dimension of the TECS-CE could be translated into practical intervention strategies for strengthening Taijiquan exercise commitment among Chinese older adults. The interview questions focused on participants’ experiences, perspectives, and recommendations regarding the development of a feasible and sustainable Taijiquan Exercise Pattern for Chinese Elderly (TEP-CE).

To minimize the influence of predetermined concepts and allow for the emergence of additional contextual factors, an additional open-ended question was included. This question encouraged participants to provide recommendations beyond the existing TECS-CE framework and to identify other factors relevant to Taijiquan participation and program implementation.

Prior to data collection, the interview guide was reviewed by five experts to establish content validity using the Index of Item-Objective Congruence (IOC). Each interview question was evaluated on a three-point scale ranging from −1 (not congruent) to 0 (uncertain) and +1 (congruent). Interview questions with IOC values of 0.50 or higher were considered acceptable. All interview questions met this criterion, indicating satisfactory content validity and appropriateness for qualitative data collection.

### 2.4 Data collection

Data collection was conducted through semi-structured, in-depth interviews with 16 purposively selected participants representing four stakeholder groups: Taijiquan administrators or managers, Taijiquan instructors or leaders, researchers or scholars, and older adult Taijiquan practitioners.

Before each interview, the researchers explained the purpose, procedures, and voluntary nature of the study to the participants. Written informed consent was obtained from all participants before data collection. Participants were informed that they could refuse to answer any question or withdraw from the study at any time without penalty.

The interviews were conducted individually in a quiet and suitable location to ensure privacy and minimize distractions. The semi-structured interview guide was used to explore the eight dimensions of the Taijiquan Exercise Commitment Scale for Chinese Elderly (TECS-CE), including want-to commitment, have-to commitment, satisfaction, social constraints, involvement alternatives, personal investment, social support, and involvement opportunities.

In addition, participants were encouraged to provide recommendations regarding contextual factors, program implementation, and practical strategies for developing the Taijiquan Exercise Pattern for Chinese Elderly (TEP-CE).

With participants’ permission, all interviews were audio-recorded and subsequently transcribed verbatim. All interviews were conducted in Mandarin Chinese and transcribed verbatim in Chinese. The interview transcripts were initially coded in the original language to preserve participants’ meanings and cultural nuances. Following data analysis, the coding results and representative quotations included in this manuscript were translated into English by the first author, who is proficient in both Mandarin Chinese and English. To enhance linguistic validity, the English translations were independently reviewed by a bilingual researcher with expertise in qualitative research and academic writing. Any discrepancies were discussed and resolved through consensus, with particular attention to preserving the conceptual meaning and cultural context of the original Chinese data rather than relying on literal translation. This translation and verification process was undertaken to enhance the linguistic accuracy, credibility, and trustworthiness of the qualitative findings. Data collection and preliminary analysis were conducted concurrently, allowing the researchers to continuously examine emerging concepts and assess whether subsequent interviews continued to generate new information relevant to the research objectives. This iterative process supported the assessment of information saturation throughout the interview process (Saunders et al., 2018).

### 2.5 Information saturation

Information saturation was assessed throughout the data collection and analysis processes. Interviews were conducted with participants representing four stakeholder groups: administrators or managers, instructors or leaders, researchers or scholars, and older adult Taijiquan practitioners. The inclusion of these groups was intended to ensure that the phenomenon was examined from management, instructional, academic, and direct participant perspectives.

Data collection continued until subsequent interviews no longer generated substantial new concepts, recommendations, or perspectives relevant to the research objectives. Saturation was assessed through continuous review and comparison of interview data across participants and stakeholder groups. The researchers examined whether subsequent interviews contributed additional concepts or primarily repeated information that had already been identified in previous interviews (Saunders et al., 2018).

The final sample comprised 16 participants. The adequacy of the sample was determined based on the richness and relevance of the information obtained, the recurrence of conceptual categories, and the extent to which the data addressed the objectives of developing the TEP-CE. This approach was consistent with the principle of information power, whereby the required sample size is influenced by the specificity of the study aim, the characteristics of the sample, the quality of the dialogue, and the analytical strategy (Malterud et al., 2016).

Because the qualitative phase aimed to generate and organize intervention components rather than estimate population parameters, sample adequacy was determined primarily by information richness, conceptual repetition, and the representation of diverse stakeholder perspectives (Malterud et al., 2016; Patton, 2015). The assessment of saturation also considered both the recurrence of concepts and the extent to which additional interviews contributed new information to the developing analytical framework (Saunders et al., 2018).

The information obtained from the final sample was considered sufficient to support the subsequent coding, categorization, and thematic synthesis described in Section 2.6.

### 2.6 Data processing and analysis

Qualitative data processing and analysis were conducted concurrently with data collection using an iterative process of data familiarization, coding, categorization, constant comparison, and interpretation. The analytical process was guided by established principles of qualitative research and qualitative thematic synthesis (Patton, 2015; Braun and Clarke, 2006).

All interview recordings were transcribed verbatim prior to analysis. The transcripts were reviewed repeatedly to ensure familiarity with the data and to identify meaningful statements related to Taijiquan exercise commitment, participation, intervention development, and contextual factors influencing exercise adherence. NVivo version 12.0 (QSR International Pty Ltd., Melbourne, Australia) was used to facilitate the organization, coding, retrieval, and management of the qualitative data.

The analytical process consisted of three sequential but iterative stages: open coding, axial coding, and selective coding. The reporting of the qualitative research was structured in accordance with the principles of transparent reporting for interview-based qualitative studies, as recommended by the Consolidated Criteria for Reporting Qualitative Research (COREQ) (Tong et al., 2007).

#### 2.6.1 Open coding

During the open-coding stage, each interview transcript was read repeatedly to obtain an overall understanding of the participants’ experiences, perceptions, and recommendations. Meaningful words, phrases, sentences, and passages relevant to the research objectives were identified and assigned initial codes.

The initial coding process focused on concepts related to the eight dimensions of the TECS-CE, including participants’ commitment to Taijiquan exercise, satisfaction with exercise participation, social constraints, alternative activities, personal investment, social support, and opportunities for involvement. Additional codes were also created for concepts that emerged from the data but were not directly represented in the original TECS-CE framework.

This process generated 80 free nodes representing the initial concepts identified from the interview data. The free nodes reflected participants’ experiences, perceptions, recommendations, and practical suggestions regarding the development and implementation of a sustainable Taijiquan exercise program for Chinese older adults.

#### 2.6.2 Axial coding

During the axial-coding stage, the 80 free nodes were reviewed and compared to identify conceptual similarities, relationships, and differences. Related codes were grouped into broader categories according to their underlying meanings and relevance to the research objectives.

This stage facilitated the identification of relationships among concepts related to Taijiquan exercise commitment, intervention strategies, social influences, environmental support, program implementation, exercise participation, and participant experiences. The categories were also compared with the theoretical dimensions of the TECS-CE to determine how the existing commitment constructs could be translated into practical intervention components.

Where several free nodes expressed similar meanings, they were integrated into broader conceptual categories. Conversely, concepts that represented distinct intervention mechanisms were retained as separate categories to preserve the richness and specificity of the participants’ perspectives.

#### 2.6.3 Selective coding

During the selective-coding stage, the categories developed during axial coding were integrated into overarching themes that explained how the eight dimensions of the TECS-CE could be translated into practical strategies for strengthening Taijiquan exercise commitment among Chinese older adults.

The researchers synthesized the emerging themes with the theoretical framework of the TECS-CE and the practical recommendations provided by the four stakeholder groups. This synthesis resulted in the identification of the principal components of the proposed Taijiquan Exercise Pattern for Chinese Elderly (TEP-CE).

The selective-coding process focused on identifying central concepts that could provide a coherent structure for the proposed intervention framework. The final themes therefore represented an integration of empirical qualitative evidence and the theoretical constructs underlying the TECS-CE.

The overall analytical process was summarized as follows:

Interview transcripts → Initial codes (free nodes) → Categories → Core themes → Components of the TEP-CE framework.

The coding process generated 80 free nodes, which were subsequently organized into conceptual categories and synthesized into the principal components of the proposed intervention framework. The final TEP-CE framework therefore represented the integration of the TECS-CE theoretical structure with the experiences, recommendations, and practical perspectives of administrators, instructors, researchers, and older adult Taijiquan practitioners.

### 2.7 Ethics statement

Ethical approval for this study was obtained from the Ethics Review Committee of Mahasarakham University, Thailand, and the Medical Research Ethics Review Committee of Henan Polytechnic University (No. 2021–36, April 4, 2021). Participation was voluntary, and all participants provided written informed consent prior to data collection.

## 3 Results

### 3.1 Outline of the expert interview

A semi-structured interview guide was developed based on the eight-factor structure of the Taijiquan Exercise Commitment Scale for Chinese Elderly (TECS-CE). The interview questions were designed to explore how each theoretical dimension of exercise commitment could be translated into practical intervention strategies for improving Taijiquan exercise commitment and exercise behavior among older adults. The interview guide consisted of nine questions:

1. Regarding the want-to commitment dimension and its items, how should the Taijiquan Exercise Pattern for Chinese Elderly (TEP-CE) be designed to improve Taijiquan exercise commitment and exercise behavior among older adults?
2. Regarding the have-to commitment dimension and its items, how should the TEP-CE be designed to improve Taijiquan exercise commitment and exercise behavior among older adults?
3. Regarding satisfaction with Taijiquan exercise and its items, how should the TEP-CE be designed to improve Taijiquan exercise commitment and exercise behavior among older adults?
4. Regarding social constraints and their items, how should the TEP-CE be designed to improve Taijiquan exercise commitment and exercise behavior among older adults?
5. Regarding involvement alternatives and their items, how should the TEP-CE be designed to improve Taijiquan exercise commitment and exercise behavior among older adults?
6. Regarding personal investment and its items, how should the TEP-CE be designed to improve Taijiquan exercise commitment and exercise behavior among older adults?
7. Regarding social support and its items, how should the TEP-CE be designed to improve Taijiquan exercise commitment and exercise behavior among older adults?
8. Regarding involvement opportunities and their items, how should the TEP-CE be designed to improve Taijiquan exercise commitment and exercise behavior among older adults?
9. What additional suggestions would you provide for developing a scientific, practical, and reasonable TEP-CE to improve Taijiquan exercise commitment and exercise behavior among older adults in China?

The interview questions were intentionally designed to combine theory-driven questions based on the TECS-CE with an open-ended question that allowed participants to identify additional contextual factors and intervention strategies not directly represented in the original theoretical framework.

### 3.2 Results of the validity test of the interview guide

To ensure that the interview questions were consistent with the research objectives and the theoretical framework of the study, the interview guide was evaluated by five experts before the commencement of data collection. The content validity of the interview questions was assessed using the Index of Item-Objective Congruence (IOC) (Rovinelli and Hambleton, 1977).

Each expert evaluated the congruence between each interview question and the study objectives using a three-point scale ranging from −1 to +1. A score of +1 indicated that the question clearly measured the intended content, 0 indicated uncertainty regarding its congruence with the intended content, and −1 indicated that the question did not measure the intended content (Rovinelli and Hambleton, 1977).

The results indicated that all interview questions met the predetermined IOC criterion of ≥0.50. The five experts therefore agreed that the questions were sufficiently congruent with the objectives and conceptual framework of the study. Accordingly, the interview guide demonstrated acceptable content validity and was considered appropriate for collecting qualitative data concerning the development of the TEP-CE.

### 3.3 Expert interview data processing and analytical results

The qualitative data obtained from the 16 expert interviews were analyzed using an iterative coding process. The analysis was conducted to establish a transparent connection between the original interview data, the initial codes, the conceptual categories, the emerging themes, and the final intervention components of the TEP-CE.

The analytical process involved four interconnected levels:

**Raw interview data → initial free nodes → conceptual categories → core themes → TEP-CE intervention components**

First, all 16 interview transcripts were imported into NVivo 12.0 and reviewed repeatedly to ensure familiarity with the data and the accuracy of the coding process. Second, meaningful words, phrases, sentences, and passages related to Taijiquan exercise commitment, participation, social influences, intervention strategies, and program implementation were identified and assigned initial codes. The open-coding process generated 80 free nodes.

Third, the free nodes were compared according to their meaning, conceptual similarity, and potential contribution to the research objectives. Related free nodes were grouped into broader conceptual categories through axial coding. For example, statements concerning verbal encouragement, daily affirmations, and expressions of personal determination were examined together as concepts related to strengthening internal motivation and want-to commitment. Similarly, statements concerning attendance monitoring, absence reminders, and recognition of regular participation were examined as potential strategies for strengthening exercise continuity and commitment through structured external support.

Fourth, the categories were compared with the eight dimensions of the TECS-CE and integrated with additional concepts emerging from the interviews. Selective coding was then used to identify the central intervention themes that could provide a coherent structure for the TEP-CE.

Thus, the 80 free nodes were not treated as independent findings. Rather, they served as the initial empirical units from which broader categories and intervention themes were developed. The frequency and recurrence of coded concepts were considered as indicators of the prominence of particular issues in the interview data, whereas the final intervention components were determined through interpretation of the meaning, relationships, and practical implications of the coded data.

#### 3.3.1 Contribution of code frequency analysis and Table 1

**Table 1:** Ranking of core words identified from expert interviews for developing the Taijiquan Exercise Pattern for Chinese Elderly (TEP-CE).

No.	Core words	*F*	No.	Core words	*F*	No.	Core words	*F*	No.	Core words	*F*
1	Taijiquan	995	21	Participant	75	41	Process	47	61	Inherited	31
2	Exercise	785	22	Develop	71	42	Group	46	62	Scientific	31
3	Elderly	496	23	Project	67	43	Opportunity	46	63	Communicate	30
4	Program	222	24	Practice	66	44	Formulate	44	64	Behavior	30
5	Society	181	25	Want to	64	45	Promote	43	65	Reasonable	30
6	Formulate	177	26	Culture	64	46	Adherence	41	66	Reduce	29
7	Entry	170	27	Personal	63	47	Content	41	67	Energy	29
8	Feel	164	28	Substitute	61	48	Healthy	40	68	Guide	29
9	Commitment	154	29	Should	58	49	Fitness	39	69	Share	28
10	Participate	140	30	Question	52	50	Improve	37	70	Have	28
11	Conduct	133	31	Factor	52	51	Consider	35	71	Explain	27
12	Promise	128	32	Aging	52	52	Encourage	34	72	Exist	26
13	Sports	125	33	Invest	52	53	Leader	34	73	Result	25
14	Practice	114	34	Influence	51	54	Environment	34	74	Produce	25
15	Intervene	100	35	Bring	50	55	Activity	34	75	Psychology	25
16	Ability	92	36	Demand	49	56	Publicity	33	76	Interview	24
17	Exerciser	91	37	Subject	49	57	Different	32	77	Government	24
18	Increase	87	38	Enjoy	48	58	Join	31	78	Related	23
19	Support	79	39	Put in	47	59	Suggest	31	79	Case	23
20	Constraint	79	40	Aspect	47	60	Value	31	80	Posture	23

This table displays the ranking core words derived from expert interviews on the TEP-CE, organized by their frequency (F). The rankings indicate the relative significance or prominence of each term as determined through the analysis of interview data. The interview analysis identified 80 frequently occurring core words. The most frequently mentioned terms were Taijiquan (*F* = 995), Exercise (*F* = 785), Elderly (*F* = 496), Program (*F* = 222), and Society (*F* = 181), indicating that participants emphasized both exercise participation and the broader social context of Taijiquan practice.

The frequency analysis of the coded interview data was used to identify concepts that appeared repeatedly across the expert interviews. Table 1 presents the frequency of the principal words and codes identified during the qualitative analysis.

The frequency information provided an initial descriptive overview of the content emphasized by participants. Concepts such as Taijiquan, exercise, older adults, commitment, participation, intervention, social support, program development, exercise adherence, and involvement opportunities appeared repeatedly in the interview data. These concepts were therefore used as important indicators during the subsequent process of categorization and thematic interpretation.

However, code frequency alone was not used to determine the final structure of the TEP-CE. Instead, frequency analysis was used as a preliminary analytical step to identify recurrent concepts and to support comparison across the interview data. The researchers subsequently returned to the original interview transcripts to examine the meaning and context in which each concept was expressed.

The analytical process therefore proceeded from the frequency of recurring concepts to the interpretation of their underlying meanings. Related codes were then grouped into conceptual categories, and these categories were compared with the eight theoretical dimensions of the TECS-CE. This process enabled the researchers to identify how participants’ recommendations could be transformed from isolated suggestions into structured intervention strategies.

Accordingly, Table 1 contributes to the thematic analysis by demonstrating the recurrence and prominence of key concepts in the interview data, while the subsequent coding and interpretation processes established the conceptual relationships necessary for developing the TEP-CE framework.

#### 3.3.2 Contribution of the word cloud to the interpretation of interview data

**Figure 2:**
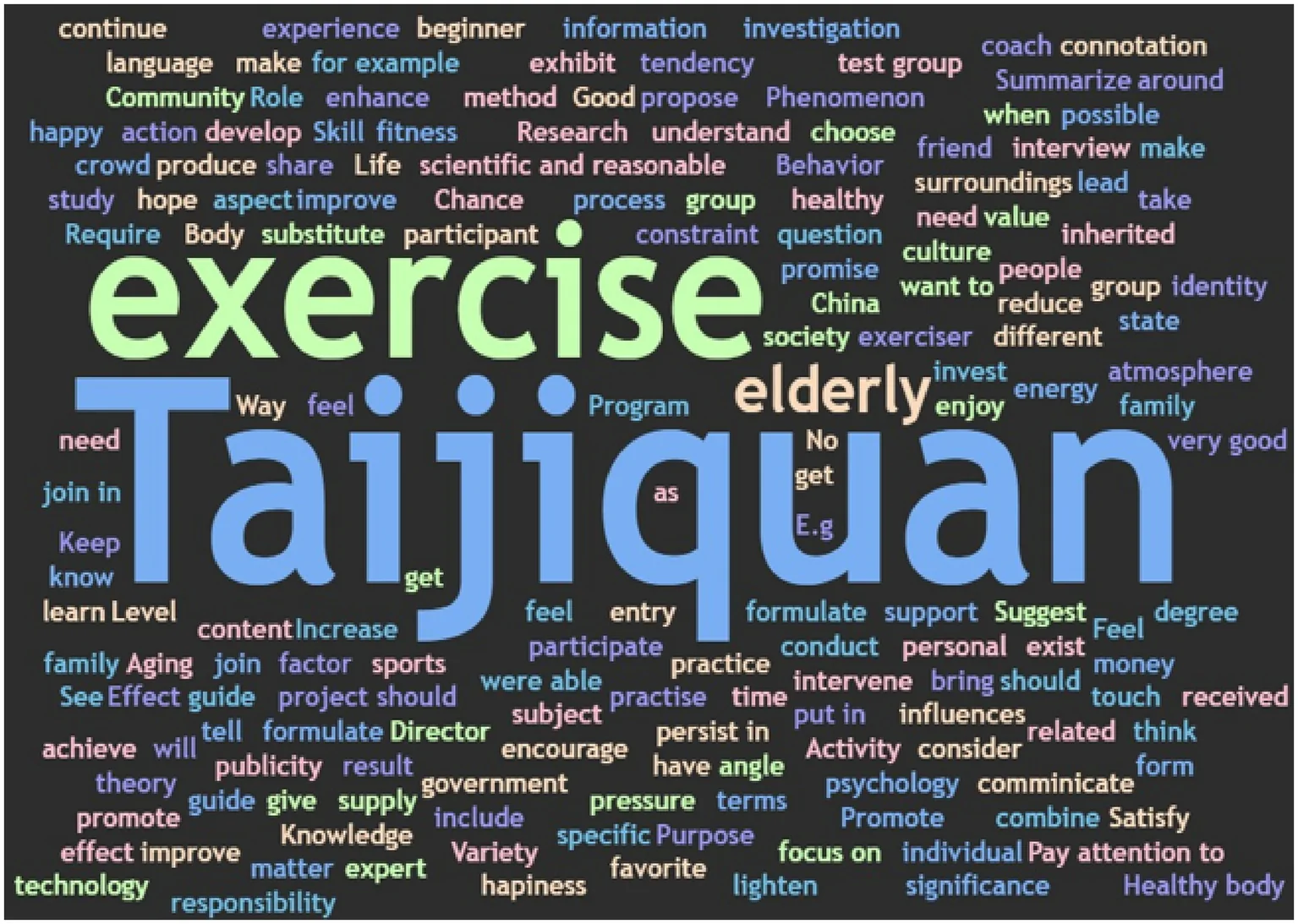
Vocabulary cloud of expert interview data on the TEP-CE.

The word cloud presented in Figure 2 provides a visual representation of the relative prominence of frequently occurring words and concepts in the expert interview data. Larger words represent concepts that occurred more frequently in the coded interview materials.

The word cloud visually demonstrates that the interview discussions were strongly centered on Taijiquan exercise, older adults, exercise participation, commitment, intervention, social interaction, and program development. Therefore, Figure 2 provides a visual overview of the dominant content of the interview data and complements the frequency information presented in Table 1.

Nevertheless, the word cloud was used as a supplementary descriptive tool rather than as an independent method of thematic analysis. The identification of the final intervention themes was based on the systematic examination of the original interview data, the coding process, the grouping of related free nodes, and the interpretation of the relationships among conceptual categories.

The integration of Table 1 and Figure 2, and the qualitative coding process provided a progressive analytical pathway from the original interview data to the final intervention framework:

**Interview transcripts → recurrent words and concepts → 80 free nodes → conceptual categories → core themes → five intervention aspects of the TEP-CE.**

This analytical pathway addressed the relationship between the rich qualitative data and the final intervention framework by demonstrating how the five intervention aspects were derived from the organization and interpretation of the interview data rather than being arbitrarily determined in advance.

### 3.4 Development of the five principal intervention aspects of the TEP-CE

Following the coding and thematic synthesis of the interview data, the researchers integrated the emerging categories with the eight dimensions of the TECS-CE. The purpose of this synthesis was to determine how the theoretical constructs of exercise commitment could be translated into practical intervention mechanisms that could be implemented in a Taijiquan exercise program for Chinese older adults.

The results indicated that the eight dimensions of the TECS-CE were interconnected and could be operationalized through five complementary intervention aspects. These five aspects were developed by comparing the meaning of the coded data, identifying relationships among intervention recommendations, and examining how different strategies could influence exercise commitment and exercise behavior.

The five principal intervention aspects of the TEP-CE were:

1. Taijiquan exercise content intervention;
2. Audio and verbal language intervention for Taijiquan exercisers;
3. Leader-to-participant intervention;
4. Mutual intervention among Taijiquan participants; and
5. Monitoring and recording of the Taijiquan exercise intervention program.

These five aspects were not considered independent components. Rather, they represented interconnected intervention mechanisms. The exercise content provided the behavioral context, audio and verbal strategies reinforced psychological commitment, leaders provided structured guidance and social influence, peer interaction strengthened social support and involvement, and monitoring and recording provided feedback and continuity mechanisms.

The relationship between the eight dimensions of the TECS-CE and the five intervention aspects of the TEP-CE, as derived from the synthesis of the qualitative interview findings, is presented in Table 2.

**Table 2:** Relationship between the TECS-CE dimensions and the five intervention aspects of the TEP-CE.

TECS-CE dimension	Main intervention mechanisms identified from the interviews	Corresponding TEP-CE intervention aspect
Want-to commitment	Daily affirmations, verbal encouragement, and self-directed commitment	Audio and verbal language intervention
Have-to commitment	Health meaning, social responsibility, and external motivation	Leader-to-participant intervention
Satisfaction	Positive feedback, recognition, and sharing of exercise benefits	Leader-to-participant and mutual participant intervention
Social constraints	Attendance records, reminders, and recognition of regular participation	Monitoring and recording intervention
Involvement alternatives	Taijiquan-related environment, cultural identity, and perceived uniqueness of Taijiquan	Taijiquan exercise content intervention
Personal investment	Additional practice, participation in events, learning materials, and skill development	Taijiquan exercise content and leader-to-participant intervention
Social support	Family, leader, and peer encouragement, together with social recognition	Leader-to-participant and mutual participant intervention
Involvement opportunities	Group interaction, social activities, demonstrations, and sharing of experiences	Mutual participant intervention and monitoring intervention

As shown in Table 2, the eight dimensions of the TECS-CE were not treated as isolated intervention targets. Rather, the qualitative analysis demonstrated that several dimensions could be addressed through shared intervention mechanisms. For example, satisfaction and social support were both linked to leader encouragement, positive feedback, and peer interaction, whereas personal investment and involvement alternatives were linked to the organization and enrichment of Taijiquan-related activities and environments. This synthesis provided the basis for consolidating the eight theoretical dimensions into five practical intervention aspects of the TEP-CE. Therefore, Table 2 represents the analytical linkage between the theoretical framework of the TECS-CE and the empirically derived intervention structure of the TEP-CE. The five intervention aspects were developed by synthesizing the 80 initial free nodes, grouping related codes into conceptual categories, and integrating these categories with the eight TECS-CE dimensions.

### 3.5 Intervention strategies for want-to commitment

The interview findings indicated that want-to commitment should be strengthened primarily through strategies that reinforce personal intention, psychological readiness, and repeated self-directed commitment to Taijiquan exercise.

Participants recommended that leaders use verbal suggestions and positive encouragement to support statements such as “I want to exercise,” “I am willing to participate,” and “I am determined to continue exercising.” These statements were considered suitable for integration into daily intervention activities.

The experts also recommended transforming selected want-to commitment items into short audio messages that participants could listen to regularly. The intention was to provide repeated exposure to positive commitment statements and reinforce participants’ personal motivation to engage in Taijiquan exercise.

In addition, participants could be encouraged to read or repeat selected want-to commitment statements at least once daily. This strategy was considered particularly appropriate because the original six items contained some conceptual similarities. The experts therefore recommended consolidating overlapping statements to make the intervention content simpler and easier to understand.

The interview findings also indicated that language with potentially negative psychological effects should be avoided. In particular, the sixth item of the want-to commitment dimension was not recommended for direct use in the language intervention because participants considered that its wording could potentially produce an undesirable psychological response.

Overall, the findings suggested that want-to commitment should be operationalized through a combination of positive verbal reinforcement, daily self-affirmation, simplified commitment statements, and repeated exposure to personally meaningful exercise intentions.

### 3.6 Intervention strategies for have-to commitment

The findings indicated that have-to commitment should be developed through meaningful explanations of the health, social, and cultural value of Taijiquan exercise rather than through coercive or psychologically threatening messages.

The experts recommended that leaders discuss the value and social significance of Taijiquan exercise with participants regularly, preferably at least once per week. The content could include health promotion, participation in national fitness activities, and the preservation of Taijiquan as an important element of traditional Chinese culture.

The experts also recommended the use of authentic and understandable examples to translate abstract commitment concepts into concrete daily meanings. For example, the idea of maintaining Taijiquan exercise could be connected with the desire to improve health, reduce the risk of illness, maintain independence, and reduce the potential caregiving burden on family members.

The interview findings further indicated that have-to commitment content should be presented carefully to avoid creating psychological discomfort. Although the concept involves a sense of obligation or necessity, the intervention should not be framed as coercion.

The experts also recommended making the content more specific and intuitive. For example, the social and cultural context of Jiaozuo City and its association with Taijiquan could be used to develop meaningful statements regarding civic identity and cultural participation.

Thus, the intervention for have-to commitment should combine health education, social and cultural meaning, authentic examples, and contextualized messages that encourage participants to perceive Taijiquan exercise as meaningful and valuable in their everyday lives.

### 3.7 Intervention strategies for satisfaction

The expert interviews indicated that satisfaction was closely related to the quality of the exercise experience, interpersonal relationships, recognition of individual progress, and participants’ perception of the benefits obtained from Taijiquan exercise.

The experts emphasized that leaders should provide high-quality services and create a positive exercise environment. The quality of the exercise group and the manner in which participants were treated were considered important contributors to satisfaction.

Participants also recommended that leaders enhance participants’ sense of achievement and happiness through positive feedback. For example, leaders could regularly recognize improvements in exercise performance, participation, physical function, or personal progress.

In addition, positive communication among participants was considered important. Mutual encouragement, praise, and care could contribute to a supportive social environment and enhance satisfaction with participation in Taijiquan exercise.

The experts also recommended publicizing the health and psychological benefits of Taijiquan exercise while providing opportunities for participants to share their own experiences. Participants could discuss changes in physical health, mental state, mood, and daily functioning. Such sharing could help participants recognize the personal benefits of continued exercise.

The findings therefore indicated that satisfaction could be strengthened through quality service, positive feedback, peer encouragement, recognition of progress, and opportunities to share perceived benefits.

### 3.8 Intervention strategies for social constraints

The findings indicated that social constraints should be incorporated into the intervention in a gentle and supportive manner rather than through strict or coercive requirements.

The experts suggested that the intervention should provide a degree of external structure that encourages regular participation while minimizing feelings of pressure or resistance. Several practical strategies were recommended, including attendance records, reminders for missed sessions, recognition of regular participation, certificates for consistent attendance, and designated exercise locations.

Importantly, the experts did not recommend mandatory attendance requirements. Instead, attendance monitoring should be used as a supportive mechanism that increases awareness of participation and encourages continuity.

The interview findings also suggested that the language used in social constraint interventions should focus primarily on the individual’s own health and well-being rather than excessive obligation to other people. The experts considered that older adults may respond more positively to messages emphasizing personal health, independence, and quality of life.

The experts also recommended consolidating similar items within this factor to simplify the intervention content.

Overall, social constraints were operationalized through soft external structure, participation monitoring, reminders, recognition, and supportive expectations rather than coercive enforcement.

### 3.9 Intervention strategies for involvement alternatives

The findings indicated that involvement alternatives should be addressed by strengthening participants’ perceived value and uniqueness of Taijiquan while creating an environment that reinforces Taijiquan-related identity and participation.

The experts suggested that the exercise environment should be designed to emphasize Taijiquan. For example, the use of Taijiquan-related images, slogans, cultural symbols, and sculptures could strengthen the visibility of Taijiquan and reinforce its cultural and exercise-related identity.

The leaders were also encouraged to explain the unique characteristics, value, and significance of Taijiquan in comparison with other forms of physical activity. These explanations should focus on the distinctive combination of physical exercise, health promotion, traditional culture, and social participation associated with Taijiquan.

The experts further recommended that leaders and participants regularly discuss the benefits, enjoyable aspects, and traditional cultural meanings of Taijiquan. Such discussions could help participants develop a stronger psychological connection with Taijiquan and reduce the likelihood that alternative activities would replace their participation.

The findings therefore indicated that intervention for involvement alternatives should focus on strengthening the perceived uniqueness, value, cultural meaning, and environmental visibility of Taijiquan.

### 3.10 Intervention strategies for personal investment

The findings indicated that personal investment should be strengthened by encouraging participants to invest additional time, energy, effort, and attention in Taijiquan beyond scheduled exercise sessions.

The experts recommended that participants be encouraged to engage in additional Taijiquan-related activities, including workshops, retreats, performances, local competitions, seminars, and other opportunities for continued learning and participation.

The experts also recommended the use of supplementary learning resources, such as Taijiquan newspapers, magazines, books, videos, and other educational materials. These resources could extend participants’ engagement with Taijiquan into their daily lives.

In addition, leaders should provide information about the theoretical foundations, exercise methods, technical characteristics, and higher-level skills of Taijiquan. The experts considered that a deeper understanding of Taijiquan could increase participants’ interest in continued learning and encourage them to invest additional effort and time in improving their practice.

Participation in performances, qualification activities, and Taijiquan-related events was also recommended as a means of expanding participants’ involvement beyond routine exercise.

Thus, personal investment was operationalized through additional practice, continued learning, participation in Taijiquan-related activities, access to educational resources, and progressive skill development.

### 3.11 Intervention strategies for social support

The findings indicated that social support should be developed through multiple sources, including leaders, peers, family members, and broader social and policy environments.

The experts recommended that leaders explain the support provided by public health and national fitness policies and communicate the broader social recognition of physical activity and Taijiquan as a means of promoting health.

At the interpersonal level, leaders should encourage participants’ exercise behavior and pay attention to their progress and outcomes. Recognition and encouragement from leaders were considered important for strengthening participants’ perceptions of support.

The experts also recommended encouraging participants to share their exercise experiences and outcomes with family members and friends. Such communication could help participants obtain support from their immediate social networks.

At the group level, mutual encouragement and peer support were considered essential. Participants could share their experiences, achievements, and perceived benefits with one another, thereby creating a supportive social environment.

Overall, the findings indicated that social support should be operationalized through policy awareness, leader encouragement, family communication, peer support, and the sharing of exercise achievements.

### 3.12 Intervention strategies for involvement opportunities

The findings indicated that involvement opportunities should extend beyond routine Taijiquan practice and provide participants with opportunities for social interaction, learning, communication, and meaningful participation.

The experts recommended group technical exchanges, demonstrations, sharing of exercise experiences, and other activities that could increase participants’ enthusiasm and enjoyment. These activities could also provide opportunities for participants to divert attention from daily stress and develop stronger social relationships.

The experts further recommended that participants be encouraged to strengthen interpersonal relationships and friendships beyond the formal exercise sessions. This could increase the social value of participation and enhance the attractiveness of continued involvement in Taijiquan.

In addition, the leaders could provide information concerning research findings related to the health-promoting effects of Taijiquan and organize supplementary recreational activities during or around exercise sessions.

The experts also recommended regular monitoring of physical indicators, such as blood pressure and heart rate, where appropriate, to help participants observe changes in physical function and exercise-related outcomes over time.

Because the involvement opportunities factor contained several overlapping concepts, the experts recommended consolidating similar intervention activities to avoid unnecessary repetition.

Overall, involvement opportunities were operationalized through technical exchange, social interaction, experience sharing, educational activities, recreational activities, and opportunities to observe individual progress.

### 3.13 Synthesis of the expert interview findings into the TEP-CE framework

The findings from the expert interviews demonstrated that the eight dimensions of the TECS-CE could be translated into five interconnected intervention aspects of the TEP-CE.

The analytical process began with the identification of 80 free nodes from the original interview data. These nodes represented specific experiences, recommendations, and intervention ideas expressed by participants. Through comparison and grouping, related nodes were organized into broader categories. These categories were then interpreted in relation to the eight theoretical dimensions of the TECS-CE.

The synthesis demonstrated that the theoretical dimensions were not isolated constructs. Instead, several dimensions could be addressed through shared intervention mechanisms. For example, want-to commitment could be strengthened through audio and verbal language intervention, whereas satisfaction and social support could be addressed through leader encouragement and mutual participant interaction. Similarly, personal investment and involvement alternatives could be influenced through the organization and enrichment of Taijiquan exercise content.

The five resulting intervention aspects were therefore:

- Taijiquan exercise content intervention, which addressed the organization, enrichment, cultural meaning, and uniqueness of Taijiquan exercise;
- Audio and verbal language intervention, which addressed internal motivation and the repeated reinforcement of positive commitment statements;
- Leader-to-participant intervention, which addressed guidance, education, feedback, encouragement, social meaning, and structured support;
- Mutual intervention among Taijiquan participants, which addressed peer encouragement, social interaction, experience sharing, and mutual support; and
- Monitoring and recording of the Taijiquan exercise intervention program, which addressed attendance, participation continuity, feedback, and the observation of exercise-related progress.

Accordingly, the five aspects of the TEP-CE were derived from the integration of the empirical interview data with the theoretical structure of the TECS-CE. The final framework therefore represented a progression from rich qualitative data to codes, from codes to conceptual categories, from categories to core themes, and from core themes to practical intervention components.

This process provided the empirical justification for the five-aspect structure of the TEP-CE and established a clear analytical link between the expert interview findings and the proposed intervention framework.

## 4 Discussion

### 4.1 Interpretation of the qualitative findings

The present study was conducted to develop an outline of the Taijiquan Exercise Pattern for Chinese Elderly (TEP-CE) based on the eight dimensions of the Taijiquan Exercise Commitment Scale for Chinese Elderly (TECS-CE). The findings demonstrate that the development of the TEP-CE was not based solely on the frequency of words or the direct opinions of the interview participants. Rather, the framework was developed through a systematic analytical process that progressed from rich interview data to initial codes, conceptual categories, thematic interpretation, and finally the synthesis of five practical intervention aspects.

The findings are consistent with the theoretical proposition that commitment is an important psychological construct underlying sustained exercise participation and behavioral persistence. Previous research has demonstrated that commitment is associated with the maintenance of physical activity and can contribute to the prediction and explanation of exercise behavior (Sun, 2012). The Sport Commitment Model further proposes that commitment is influenced by multiple factors, including satisfaction, involvement alternatives, personal investment, social constraints, social support, and involvement opportunities (Scanlan et al., 1993; Wilson et al., 2004; Waldron and Troupe, 2008). These theoretical dimensions provided an important conceptual foundation for interpreting the qualitative data in the present study.

The present findings are also consistent with recent evidence indicating that sustained exercise participation among older adults is influenced by multiple interacting determinants rather than by physical benefits alone. Recent systematic reviews have shown that long-term exercise adherence is strengthened when interventions incorporate motivational support, social engagement, individualized exercise planning, instructor guidance, and behavioral reinforcement (Wu et al., 2025; Shaw et al., 2022). Likewise, contemporary evidence on Tai Chi interventions suggests that social interaction, personalized support, and meaningful engagement contribute substantially to maintaining long-term participation and maximizing health-related outcomes among older adults (Koren et al., 2021; Leung et al., 2022; Fan et al., 2025). These findings support the present qualitative results, which demonstrate that exercise commitment can be strengthened through an integrated intervention framework that simultaneously addresses psychological, social, behavioral, and contextual factors. Rather than viewing commitment as a single psychological attribute, the present study illustrates how multiple commitment-related dimensions can be translated into practical intervention strategies for promoting sustained participation in Taijiquan among Chinese older adults.

The analytical process was conducted through several stages. First, the interview transcripts provided the primary source of rich qualitative information. The transcripts contained participants’ experiences, recommendations, explanations, and practical suggestions concerning Taijiquan participation among older adults. Second, the researchers conducted open coding and generated 80 free nodes representing meaningful concepts extracted from the interview data. Third, related free nodes were compared and grouped into broader conceptual categories through axial coding. Finally, the categories were integrated into core themes through selective coding and synthesized with the eight-factor structure of the TECS-CE.

This analytical sequence is important because the five intervention aspects were not arbitrarily determined by the researchers. Rather, they emerged from the relationships among the qualitative codes and the theoretical dimensions of exercise commitment. The process therefore followed the analytical sequence:

**Rich interview data → Initial codes/free nodes → Conceptual categories → Core themes → Five TEP-CE intervention aspects.**

This approach is consistent with established qualitative approaches to coding and thematic analysis, in which researchers move from detailed descriptions of participants’ experiences toward increasingly abstract categories and themes (Patton, 2015; Braun and Clarke, 2006; Maher et al., 2018; Azeem et al., 2012). The use of qualitative data analysis software further facilitated the systematic organization, retrieval, and comparison of coded data (Azeem et al., 2012; Paulus et al., 2017).

### 4.2 Contribution of the word-frequency analysis and word cloud to the thematic analysis

Table 1 and Figure 2 provide complementary descriptive evidence concerning the content of the interview data. Table 1 presents the frequency of frequently occurring words or coded concepts, whereas Figure 2 provides a visual representation of the relative prominence of vocabulary appearing in the interview corpus. These analyses were used as supplementary analytical tools rather than as independent evidence for determining the final themes.

The frequency analysis helped identify recurring concepts that appeared repeatedly across the interview data. These concepts included Taijiquan, exercise, older adults, commitment, participation, intervention, social support, exercise opportunities, and program development. The recurrence of these concepts indicated that the interview data were concentrated around several interconnected areas: the motivation to participate, the maintenance of exercise behavior, the social environment surrounding exercise, and the practical organization of Taijiquan activities.

However, word frequency alone cannot explain the meaning or relationships among concepts. Therefore, the frequently occurring words shown in Table 1 were examined in their original textual contexts and were subsequently interpreted through the coding process. A frequently occurring word was not automatically treated as a theme. Instead, the researchers returned to the original interview passages to examine how participants used the concept, what meaning it conveyed, and how it related to the theoretical dimensions of the TECS-CE.

Figure 2 provides a visual overview of the dominant vocabulary within the interview corpus. The word cloud therefore contributes to the thematic analysis by providing an initial visual representation of the semantic concentration of the data. The prominence of terms associated with Taijiquan, exercise, commitment, participation, support, intervention, and older adults reflects the central focus of the interview discussions. Nevertheless, the word cloud was used primarily to support data familiarization and to illustrate the overall content structure of the interviews. The final themes and intervention aspects were established through systematic coding, constant comparison, interpretation of contextual meaning, and theoretical synthesis rather than through word frequency alone.

Accordingly, Table 1 and Figure 2 should be understood as descriptive and supportive components of the analysis. The principal analytical evidence for the development of the TEP-CE was derived from the coded interview data and the subsequent relationships identified among free nodes, categories, and themes. This distinction strengthens the methodological rigor of the study and avoids interpreting vocabulary frequency as equivalent to thematic importance.

### 4.3 From the eight TECS-CE dimensions to five TEP-CE intervention aspects

The qualitative findings demonstrate that the five intervention aspects of the TEP-CE were developed through the integration of the empirical interview data with the theoretical structure of the TECS-CE. The five aspects were not derived simply by counting frequently occurring words or by assigning one intervention component to each TECS-CE dimension. Instead, the intervention aspects were developed by examining the conceptual relationships among the 80 initial free nodes, grouping related codes into broader categories, and interpreting how these categories could be translated into practical mechanisms for strengthening Taijiquan exercise commitment among Chinese older adults.

The findings indicate that the eight TECS-CE dimensions are theoretically distinguishable but practically interconnected. Consequently, several dimensions were found to operate through similar intervention mechanisms and could be integrated into broader intervention aspects. This process resulted in five practical intervention aspects: (1) Taijiquan exercise content intervention, (2) audio and verbal language intervention, (3) leader-to-participant intervention, (4) mutual participant intervention, and (5) monitoring and recording intervention.

The first intervention aspect concerns Taijiquan exercise content intervention. This aspect was primarily derived from the qualitative categories associated with involvement alternatives and personal investment. Participants emphasized the importance of making Taijiquan meaningful, distinctive, and attractive in comparison with other possible activities. The interview data indicated that the exercise environment, cultural identity, Taijiquan-related materials, demonstrations, technical learning, and opportunities to participate in Taijiquan-related activities could enhance participants’ perceptions of the uniqueness and value of Taijiquan.

This finding is consistent with the Sport Commitment Model, which proposes that involvement alternatives may influence commitment when individuals perceive other activities as attractive alternatives to their current activity (Scanlan et al., 1993; Wilson et al., 2004). The interview findings suggest that one strategy for addressing this issue is not necessarily to prevent older adults from engaging in other activities, but rather to strengthen the perceived value, meaning, and distinctiveness of Taijiquan. At the same time, personal investment may be strengthened when participants devote additional time, effort, energy, and resources to the activity. Additional practice, participation in Taijiquan events, learning materials, and opportunities for technical development may therefore contribute to a stronger psychological connection with Taijiquan.

The second intervention aspect concerns audio and verbal language intervention. This aspect was primarily derived from the want-to commitment dimension. The interview findings indicated that participants could be encouraged to repeatedly express, listen to, or reflect upon statements that reinforce personal willingness and determination to practice Taijiquan. Examples included statements expressing the desire to exercise, determination to continue practicing, and personal commitment to maintaining participation.

The importance of this intervention aspect lies in its potential to transform an abstract psychological construct into a repeated daily behavioral and cognitive experience. Rather than treating want-to commitment solely as a measurement construct, the findings suggest that commitment-related language may be incorporated into daily intervention activities through verbal encouragement, audio recordings, and self-directed affirmations. Such strategies may help reinforce internally motivated commitment and maintain the intention to continue exercising.

The third intervention aspect concerns leader-to-participant intervention. This aspect incorporated the qualitative findings related to have-to commitment, satisfaction, personal investment, and social support. The interview data indicated that leaders play a central role in shaping how participants understand the health value, social meaning, and personal importance of Taijiquan exercise.

However, the findings suggest that the role of leaders should extend beyond the provision of technical instruction. Leaders may explain the health benefits and social significance of Taijiquan, provide positive feedback, recognize individual progress, encourage continued participation, recommend Taijiquan-related learning opportunities, and communicate information about social and policy support for physical activity. These functions may influence both the cognitive and emotional dimensions of exercise commitment.

The findings related to satisfaction are particularly important. Satisfaction may be strengthened when participants perceive that their participation is meaningful, enjoyable, and associated with positive outcomes. Positive feedback from leaders, recognition of progress, and opportunities to share improvements in health, mood, and daily functioning may therefore reinforce the perceived benefits of Taijiquan participation. This interpretation is consistent with previous research indicating that enjoyment and satisfaction are associated with continued involvement in physical activity (Scanlan et al., 1993).

The fourth intervention aspect concerns mutual participant intervention. This aspect was primarily derived from the qualitative categories related to social support, satisfaction, and involvement opportunities. Participants emphasized the importance of mutual encouragement, positive communication, sharing of exercise experiences, mutual care, and the development of interpersonal relationships.

This finding highlights the social dimension of Taijiquan participation among older adults. Exercise may become more sustainable when it provides opportunities for social interaction and meaningful relationships. In this context, Taijiquan is not only a form of physical activity but may also function as a socially and culturally meaningful activity. Group practice, demonstrations, shared experiences, mutual encouragement, and social activities may therefore strengthen the interpersonal benefits of participation and contribute to continued commitment.

The fifth intervention aspect concerns monitoring and recording intervention. This aspect was primarily associated with social constraints and, to some extent, involvement opportunities. The interview findings suggested that attendance records, reminders, recognition of regular participation, and monitoring of selected physical indicators could provide a structured mechanism for supporting continued participation.

Importantly, the qualitative findings did not support the use of excessively coercive or punitive forms of social constraint. Instead, participants recommended supportive mechanisms, including attendance monitoring, absence reminders, participation recognition, certificates, and other forms of gentle external structure. These findings suggest that social constraints may contribute positively to exercise commitment when they are implemented in a supportive and non-threatening manner.

Taken together, the five intervention aspects demonstrate how the eight psychological dimensions of the TECS-CE can be translated into practical intervention channels. The qualitative findings indicate that commitment can be addressed through the content of the exercise itself, the language used to reinforce commitment, the behavior of leaders, interactions among participants, and systems for monitoring and recording participation. The five aspects therefore provide a practical organizational structure for the TEP-CE while retaining the theoretical foundation of the TECS-CE.

Importantly, the five intervention aspects should not be interpreted as completely independent components. Rather, they are interconnected mechanisms that may operate simultaneously. For example, an exercise activity may increase personal investment while also creating opportunities for social support; leader encouragement may enhance satisfaction while reinforcing both want-to and have-to commitment; and monitoring may provide external structure while creating opportunities for recognition and continued participation. This interconnectedness reflects the multidimensional nature of exercise commitment and provides a theoretical basis for developing flexible and individualized Taijiquan interventions for older adults.

### 4.4 Theoretical implications

The present study contributes to the literature by extending the application of the Sport Commitment Model to the development of a practical Taijiquan exercise intervention framework for older adults. Previous studies have primarily used commitment constructs to explain or predict exercise participation (Wilson et al., 2004; Creswell and Poth, 2016). The present study advances this perspective by examining how the underlying dimensions of commitment can be operationalized as intervention mechanisms.

The findings indicate that the eight TECS-CE dimensions are conceptually distinct but practically interconnected. Want-to commitment can be addressed through self-directed and verbal reinforcement. Have-to commitment can be influenced through health meaning and social responsibility. Satisfaction can be strengthened through positive feedback and recognition. Social constraints can be operationalized through supportive monitoring mechanisms. Involvement alternatives can be addressed through the strengthening of Taijiquan’s perceived uniqueness and cultural value. Personal investment can be increased through additional practice and learning opportunities. Social support can be strengthened through family, leader, and peer involvement, while involvement opportunities can be promoted through group interaction and social activities.

This integration provides a conceptual explanation of how psychological commitment may be translated into practical intervention components. The TEP-CE therefore represents a theoretically informed framework rather than a collection of independent exercise strategies.

The findings are also consistent with evidence indicating that Taijiquan provides multiple physical, psychological, cognitive, and social benefits for older adults. Previous studies have associated Tai Chi with improvements in psychological well-being, cognitive function, balance, and fall prevention (Li et al., 2001; Li, 2016; Abbott and Lavretsky, 2013; Wang et al., 2014; Tao et al., 2016; Lo and Lee, 2014; Stevens et al., 2014; Tong et al., 2007). Furthermore, earlier research has suggested that sport commitment is positively associated with exercise adherence (Rovinelli and Hambleton, 1977), while more recent systematic reviews have demonstrated that Tai Chi interventions can enhance physical function, social interaction, and overall quality of life, particularly when participation is maintained over time (Koren et al., 2021; Leung et al., 2022; Fan et al., 2025). These benefits may contribute to greater satisfaction and personal investment, thereby reinforcing exercise commitment and supporting long-term participation among older adults.

The relationship between perceived benefits and commitment is particularly important. If older adults perceive that Taijiquan contributes to improved health, balance, psychological well-being, or social interaction, these experiences may reinforce satisfaction and strengthen continued participation. In this sense, the health benefits of Taijiquan may function not only as outcomes of participation but also as mechanisms that support continued exercise commitment.

### 4.5 Practical implications

The findings provide several practical implications for the development and implementation of Taijiquan exercise programs for older adults.

First, Taijiquan programs should not focus exclusively on the technical content of exercise. Although exercise instruction remains important, the findings indicate that commitment may also be influenced by communication, social relationships, personal meaning, and monitoring systems.

Second, instructors and program leaders should adapt intervention strategies according to the psychological and social needs of participants. For example, participants with weak internal motivation may benefit from verbal encouragement and daily affirmation, whereas participants who require stronger social engagement may benefit from group interaction and peer support.

Third, exercise programs should create opportunities for participants to recognize the benefits of Taijiquan. Participants may be encouraged to share changes in their health, mood, physical function, and daily well-being. Such experiences may increase satisfaction and reinforce the perceived value of continued participation.

Fourth, monitoring systems should be implemented in a supportive manner. Attendance records, reminders, recognition of regular participation, and simple health monitoring may help participants maintain exercise routines without creating excessive psychological pressure.

Finally, the TEP-CE provides a foundation for developing individualized intervention strategies. The five intervention aspects can be combined and adjusted according to the characteristics of specific groups, including age, physical ability, exercise experience, social environment, and psychological commitment.

## 5 Conclusion

The present study developed an outline of the Taijiquan Exercise Pattern for Chinese Elderly (TEP-CE) through a qualitative analysis of interviews with Taijiquan administrators or managers, instructors or leaders, researchers or scholars, and older adult Taijiquan practitioners. The development process was theoretically guided by the eight dimensions of the Taijiquan Exercise Commitment Scale for Chinese Elderly (TECS-CE).

The findings demonstrate that the TEP-CE was developed through a systematic analytical process involving the progression from rich interview data to initial codes, conceptual categories, core themes, and practical intervention components. The 80 free nodes generated during the coding process provided the empirical foundation for identifying relationships among participants’ experiences, perceptions, and recommendations. The frequency analysis presented in Table 1 and the word cloud shown in Figure 2 supported data familiarization and illustrated the dominant vocabulary of the interview corpus, while the final intervention framework was established through contextual coding, comparison, thematic interpretation, and theoretical synthesis.

The analysis resulted in five principal intervention aspects: (1) Taijiquan exercise content intervention; (2) audio and verbal language intervention; (3) leader-to-participant intervention; (4) mutual participant intervention; and (5) monitoring and recording intervention. These five aspects provide a practical structure through which the eight dimensions of the TECS-CE may be translated into strategies for strengthening exercise commitment and supporting continued participation among older adults.

However, the present study developed an intervention framework rather than a fully standardized intervention program. Therefore, the TEP-CE should be regarded as a theoretically informed and empirically grounded framework that requires further testing in practical settings. The framework may be adapted according to the characteristics of instructors, participants, exercise environments, and local social contexts.

The findings further suggest that effective Taijiquan interventions should not be limited to physical exercise instruction. Rather, long-term participation may be supported through the integration of exercise content, internal motivation, verbal encouragement, leader support, peer relationships, social opportunities, and supportive monitoring. The TEP-CE therefore provides a foundation for the future development of more individualized and context-sensitive Taijiquan exercise programs for older adults in China.

## 6 Future research agenda

Future research should further examine the practical application, effectiveness, and adaptability of the TEP-CE across different populations and settings.

First, future studies should develop and test a comprehensive intervention manual based on the five TEP-CE intervention aspects and the eight dimensions of the TECS-CE. The manual should provide practical guidance for instructors, program leaders, and participants while allowing sufficient flexibility for adaptation to individual needs.

Second, future research should examine differences in exercise commitment and intervention needs across different age bands. Older adults are not a homogeneous population, and individuals in their 40s, 50s, 60s, 70s, 80s, and 90s may differ in physical function, health status, social roles, exercise preferences, and perceptions of successful aging. Comparative studies across age groups may therefore clarify whether different age cohorts require different motivational strategies and intervention components.

Third, future studies should compare older adults living in urban and rural environments. Differences in access to exercise facilities, social networks, cultural traditions, health services, transportation, and community resources may influence both exercise commitment and adherence. Comparative research could identify similarities and differences in the motivational factors, barriers, social constraints, and support systems influencing Taijiquan participation.

Fourth, longitudinal and intervention studies should examine whether the TEP-CE can improve exercise commitment, adherence, physical function, psychological well-being, and quality of life. Experimental studies could compare the TEP-CE with conventional Taijiquan instruction and examine whether the integration of commitment-based intervention strategies produces additional benefits.

Fifth, future research should investigate the feasibility of personalized TEP-CE interventions. Because older adults differ in their motivational profiles, social circumstances, physical abilities, and psychological needs, personalized intervention strategies may be more effective than a uniform program. The eight dimensions of the TECS-CE could potentially be used to identify individual commitment profiles and guide the selection of appropriate intervention strategies.

Finally, future research should examine the implementation of the TEP-CE across different community, institutional, clinical, and recreational settings. Such research could identify contextual factors that facilitate or hinder implementation and could support the development of policy recommendations for active aging and community-based physical activity promotion.

Comparative research across age bands and living environments may ultimately contribute to a more precise understanding of how motivational factors, commitment, social support, and barriers to exercise adherence vary among different groups of older adults. This knowledge could support more targeted policies, planning strategies, and exercise programs tailored to the specific characteristics of different populations and places of aging.

## Data Availability

The original contributions presented in the study are included in the article/Supplementary material, further inquiries can be directed to the corresponding author/s.
