# Supplementary material for: Developing a Taijiquan exercise pattern for Chinese older adults: a qualitative study based on the exercise commitment model
Source: Front Psychol. 2026 Sep 7;17:1883642. doi: 10.3389/fpsyg.2026.1883642 (PMC13592214; doi:10.3389/fpsyg.2026.1883642)
Supplement: Supplementary file 1 [file Supplementary_file_1.pdf]

Supplementary Material

Appendix A. Commitment Constructs and Subscale Items of the Taijiquan Exercise Commitment Scale for Chinese Elderly (TECS-CE).

Commitment constructs and subscale items of Taijiquan exercise

Want to Commitment

1. I am determined to keep exercising Taijiquan.

2. I am dedicated to keep exercising Taijiquan.

3. I am committed to keep exercising Taijiquan.

4. I am willing to do almost anything to keep exercising Taijiquan.

5. I want to keep exercising Taijiquan.

6. It would be hard for me to quit exercising Taijiquan.

Have to Commitment

7. I feel obligated to continue exercising Taijiquan.

8. I feel it is necessary for me to continue exercising Taijiquan.

9. I feel exercise Taijiquan is a duty.

Satisfaction

10. All things considered, exercise Taijiquan is very satisfying.

11. Because I exercise Taijiquan, I feel satisfied.

12. I find exercising Taijiquan to be very rewarding.

Social Constraints

13. People will think I am a quitter if I stop exercising Taijiquan.

14. I feel pressure from other people to exercise Taijiquan.

15. I must keep exercising Taijiquan to please others.

16. People will be disappointed with me if I quit exercising Taijiquan.

Involvement Alternatives

17. Compared to exercising Taijiquan, there are other things I could do which would be more fun.

18. Compared to exercising Taijiquan, there are other things I could do which would be more enjoyable.

19. Compared to exercising Taijiquan, there are other things I could do which would be more worthwhile.

20. I would be happier doing something else instead of exercising Taijiquan.

21. I would like to do something else instead of exercising Taijiquan.

Personal Investment

22. I have invested a lot of effort into exercising Taijiquan.

23. I have invested a lot of energy into exercising Taijiquan.

24. I have invested a lot of time into exercising Taijiquan.

Social Support

25. People important to me support my exercising Taijiquan.

26. People important to me think it is okay to exercise Taijiquan.

27. People important to me encourage me to exercise Taijiquan.

Involvement Opportunities

28. Exercising Taijiquan gives me the opportunity to feel excited.

29. Exercising Taijiquan gives me the opportunity to relieve any stress I am feeling.

30. Exercising Taijiquan gives me the opportunity to have a good time.

31. Exercising Taijiquan gives me the opportunity to be with my friends.

32. Exercising Taijiquan gives me the opportunity to improve my health and fitness.

33. Exercising Taijiquan gives me the opportunity to improve my physical skills.
