# Supplementary material for: Developing a Taijiquan exercise pattern for Chinese older adults: a qualitative study based on the exercise commitment model
Source: Front Psychol. 2026 Sep 7;17:1883642. doi: 10.3389/fpsyg.2026.1883642 (PMC13592214; doi:10.3389/fpsyg.2026.1883642)
Supplement: Supplementary file 2 [file Supplementary_file_2.pdf]

Supplementary Material

Table 1 Ranking of Core Words Identified from Expert Interviews for Developing the Taijiquan Exercise Pattern for Chinese Elderly (TEP-CE).

No.	Core words	F	No.	Core words	F	No.	Core words	F	No.	Core words	F
1	Taijiquan	995	21	Participant	75	41	Process	47	61	Inherited	31
2	Exercise	785	22	Develop	71	42	Group	46	62	Scientific	31
3	Elderly	496	23	Project	67	43	Opportunity	46	63	Communicate	30
4	Program	222	24	Practice	66	44	Formulate	44	64	Behavior	30
5	Society	181	25	Want to	64	45	Promote	43	65	Reasonable	30
6	Formulate	177	26	Culture	64	46	Adherence	41	66	Reduce	29
7	Entry	170	27	Personal	63	47	Content	41	67	Energy	29
8	Feel	164	28	Substitute	61	48	Healthy	40	68	Guide	29
9	Commitment	154	29	Should	58	49	Fitness	39	69	Share	28
10	Participate	140	30	Question	52	50	Improve	37	70	Have	28
11	Conduct	133	31	Factor	52	51	Consider	35	71	Explain	27
12	Promise	128	32	Aging	52	52	Encourage	34	72	Exist	26
13	Sports	125	33	Invest	52	53	Leader	34	73	Result	25
14	Practice	114	34	Influence	51	54	Environment	34	74	Produce	25
15	Intervene	100	35	Bring	50	55	Activity	34	75	Psychology	25
16	Ability	92	36	Demand	49	56	Publicity	33	76	Interview	24
17	Exerciser	91	37	Subject	49	57	Different	32	77	Government	24
18	Increase	87	38	Enjoy	48	58	Join	31	78	Related	23
19	Support	79	39	Put in	47	59	Suggest	31	79	Case	23
20	Constraint	79	40	Aspect	47	60	Value	31	80	Posture	23

Note: This table displays the ranking core words derived from expert interviews on the TEP-CE, organized by their frequency (F). The rankings indicate the relative significance or prominence of each term as determined through the analysis of interview data.

The interview analysis identified 80 frequently occurring core words. The most frequently mentioned terms were Taijiquan (F = 995), Exercise (F = 785), Elderly (F = 496), Program (F = 222), and Society (F = 181), indicating that participants emphasized both exercise participation and the broader social context of Taijiquan practice.

Table 2 Relationship between the TECS-CE dimensions and the five intervention aspects of the TEP-CE

TECS-CE dimension	Main intervention mechanisms identified from the interviews	Corresponding TEP-CE intervention aspect
Want-to commitment	Daily affirmations, verbal encouragement, and self-directed commitment	Audio and verbal language intervention
Have-to commitment	Health meaning, social responsibility, and external motivation	Leader-to-participant intervention
Satisfaction	Positive feedback, recognition, and sharing of exercise benefits	Leader-to-participant and mutual participant intervention
Social constraints	Attendance records, reminders, and recognition of regular participation	Monitoring and recording intervention
Involvement alternatives	Taijiquan-related environment, cultural identity, and perceived uniqueness of Taijiquan	Taijiquan exercise content intervention
Personal investment	Additional practice, participation in events, learning materials, and skill development	Taijiquan exercise content and leader-to-participant intervention
Social support	Family, leader, and peer encouragement, together with social recognition	Leader-to-participant and mutual participant intervention
Involvement opportunities	Group interaction, social activities, demonstrations, and sharing of experiences	Mutual participant intervention and monitoring intervention

As shown in Table 2, the eight dimensions of the TECS-CE were not treated as isolated intervention targets. Rather, the qualitative analysis demonstrated that several dimensions could be addressed through shared intervention mechanisms. For example, satisfaction and social support were both linked to leader encouragement, positive feedback, and peer interaction, whereas personal investment and involvement alternatives were linked to the organization and enrichment of Taijiquan-related activities and environments. This synthesis provided the basis for consolidating the eight theoretical dimensions into five practical intervention aspects of the TEP-CE.

Therefore, Table 2 represents the analytical linkage between the theoretical framework of the TECS-CE and the empirically derived intervention structure of the TEP-CE. The five intervention aspects were developed by synthesizing the 80 initial free nodes, grouping related codes into conceptual categories, and integrating these categories with the eight TECS-CE dimensions.

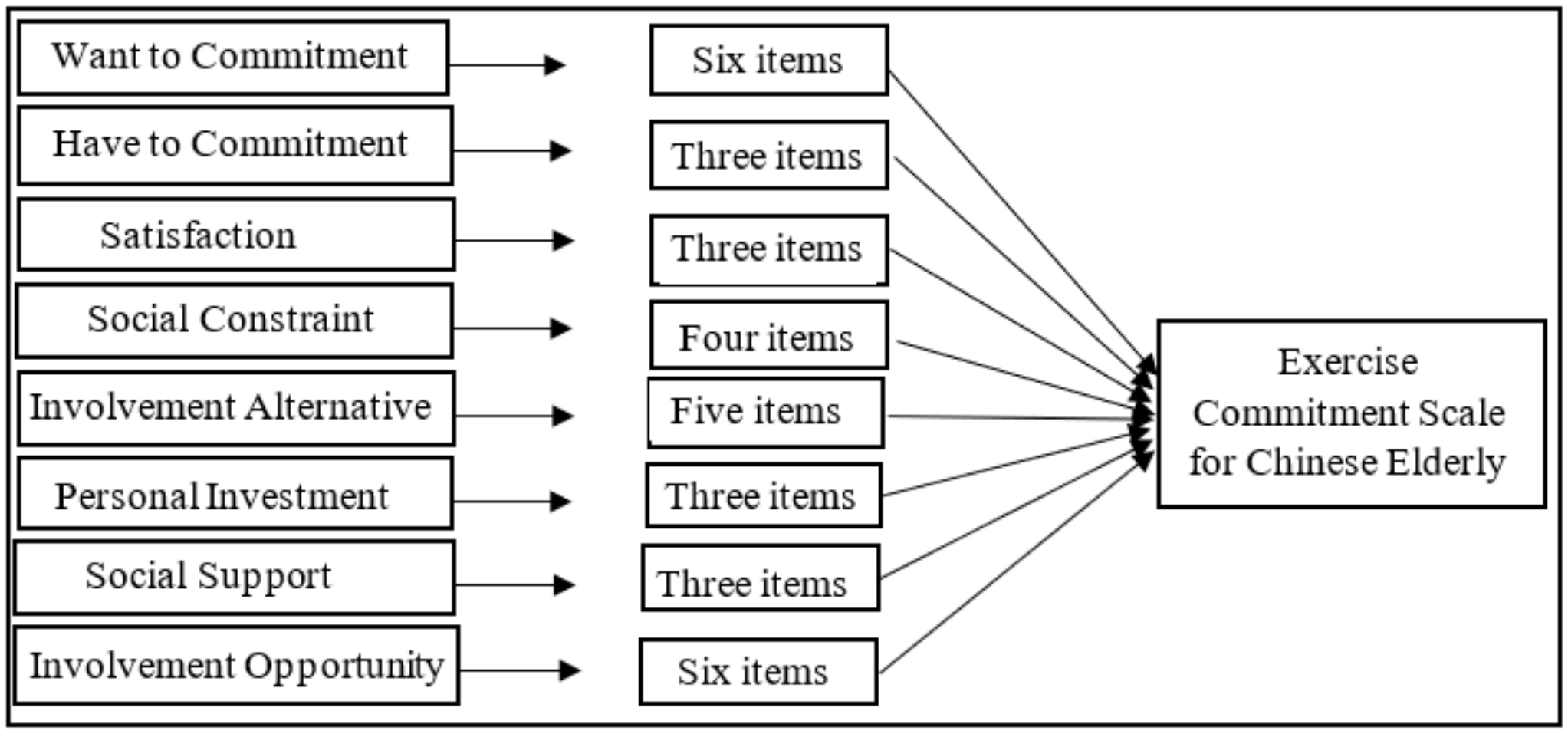

Figure 1. Conceptual Framework of the Taijiquan Exercise Commitment Scale for Chinese Elderly (TECS-CE).

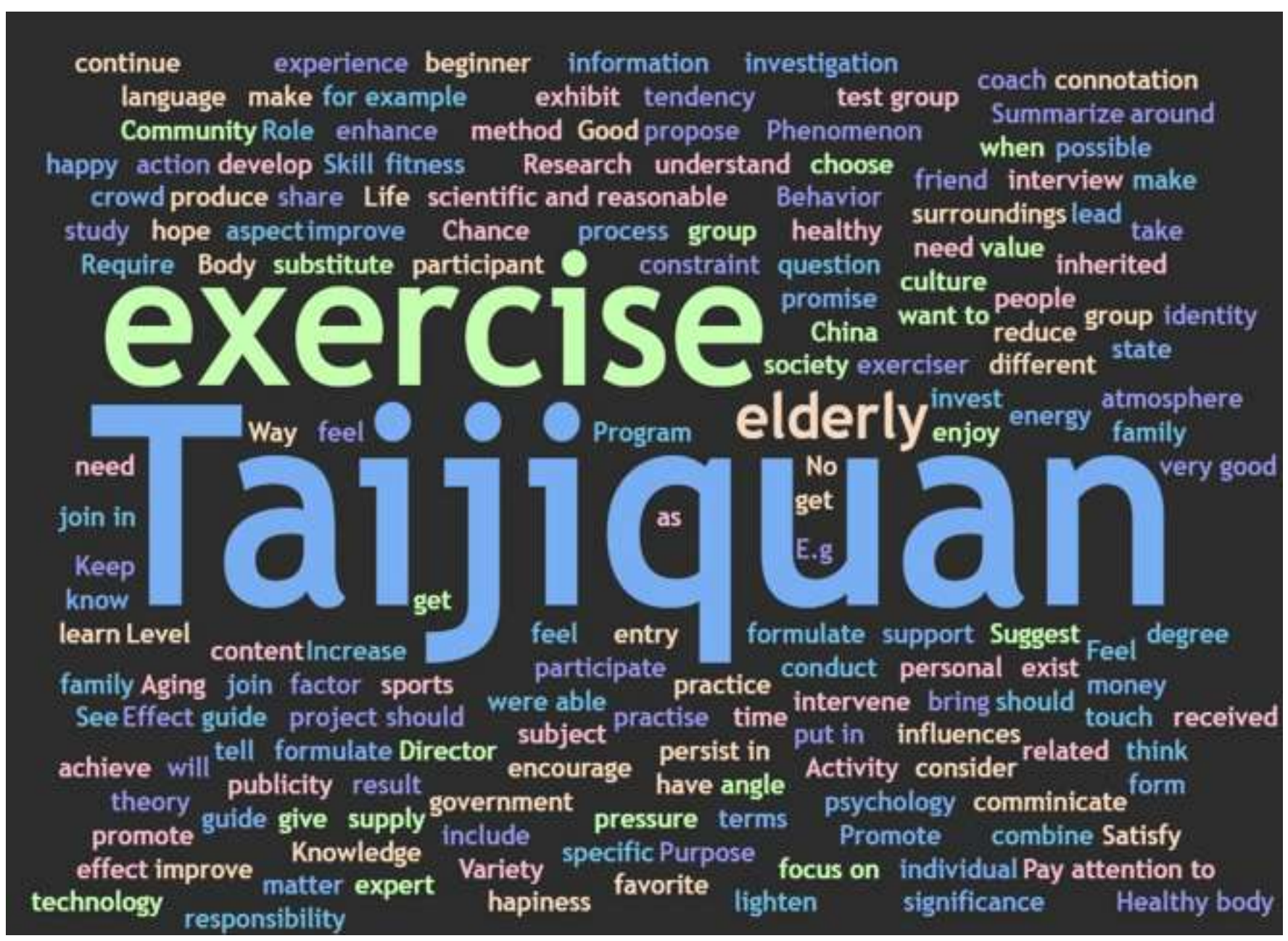

Figure 2. Vocabulary cloud of expert interview data on the TEP-CE
